# Measles Population Immunity in Hunan, China: A Serological Assessment

**DOI:** 10.1093/ofid/ofaf216

**Published:** 2025-04-08

**Authors:** Yanpeng Wu, Qianli Wang, Wei Wang, Xiaoyu Zhou, Juanjuan Zhang, Sihong Zhao, Yuxia Liang, Pengfei Wang, Filippo Trentini, Marco Ajelli, Hongjie Yu

**Affiliations:** Shanghai Institute of Infectious Disease and Biosecurity, Fudan University, Shanghai, China; Shanghai Institute of Infectious Disease and Biosecurity, Fudan University, Shanghai, China; School of Public Health, Fudan University, Key Laboratory of Public Health Safety, Ministry of Education, Shanghai, China; Shanghai Institute of Infectious Disease and Biosecurity, Fudan University, Shanghai, China; School of Public Health, Fudan University, Key Laboratory of Public Health Safety, Ministry of Education, Shanghai, China; School of Public Health, Fudan University, Key Laboratory of Public Health Safety, Ministry of Education, Shanghai, China; School of Public Health, Fudan University, Key Laboratory of Public Health Safety, Ministry of Education, Shanghai, China; Shanghai Institute of Infectious Disease and Biosecurity, Fudan University, Shanghai, China; Shanghai Pudong Hospital, Fudan University Pudong Medical Center, State Key Laboratory of Genetic Engineering, MOE Engineering Research Center of Gene Technology, School of Life Sciences, Fudan University, Shanghai, China; Dondena Centre for Research on Social Dynamics and Public Policy, Department of Decision Sciences, Bocconi University, Milan, Italy; Laboratory for Computational Epidemiology and Public Health, Department of Epidemiology and Biostatistics, Indiana University School of Public Health, Bloomington, Indiana, USA; Shanghai Institute of Infectious Disease and Biosecurity, Fudan University, Shanghai, China; School of Public Health, Fudan University, Key Laboratory of Public Health Safety, Ministry of Education, Shanghai, China; Department of Infectious Diseases, Huashan Hospital, Fudan University, Shanghai, China

**Keywords:** age-specific, antibody concentrations, contact, measles, seroprevalence

## Abstract

**Background:**

Assessing the measles immunity profile is critical for developing effective nationwide or regionwide supplementary immunization activities (SIAs). This study aims to assess measles population immunity levels in China and investigate factors contributing to age-specific heterogeneities.

**Methods:**

We conducted a cross-sectional population-based serological study in southern China between June and October 2021. We determined the population mean antibody concentration and seroprevalence by age and over time, along with their associated determinants. Moreover, we estimated the contact-adjusted immunity levels by considering both age-specific immunity levels and population contact rates.

**Results:**

Among the 1015 study participants (aged 0–95 years), the overall seroprevalence was estimated at 80.9% (95% confidence interval [CI], 78.3–83.3). When adjusting for the age-specific contact rates, the contact-adjusted immunity was estimated at 66.8% (95% CI, 56.6–75.1). Individuals younger than age 30 years showed significantly lower antibody concentration and seroprevalence (mean log concentration: 5.9, seroprevalence: 73.6% [95% CI, 69.9–77.3]) compared to those older than age 50 years (mean log concentration: 6.8, seroprevalence: 96.8% [95% CI, 94.7–98.9]). In particular, individuals born after the last SIA (2010) showed a significantly faster waning of immunity.

**Conclusions:**

Our findings highlight that immunity levels in the general population remain below the 95% threshold, underscoring the need for continued monitoring of immunity dynamics, especially for individuals born in a near-elimination setting and without subsequent SIAs.

## BACKGROUND

Measles is a highly contagious but vaccine-preventable infectious disease caused by the measles virus [1]. Children younger than age 5 years are more susceptible to measles virus infection and severe complications, as are adults, pregnant women, and malnourished or immunocompromised people. While a highly effective vaccine was introduced in the 1960s, measles still causes considerable morbidity and mortality worldwide, with an estimated 0.25 million cases reported annually between 2010 and 2017 [2]. The number of measles cases substantially increased since 2018–2019 (1.2 million cases) because of growing vaccine hesitancy, suboptimal immunity postvaccination, and inequitable immunization services in many low- and middle-income countries associated with political instability [3–5]. Disruptions to routine measles immunization services caused by the COVID-19 pandemic have further exacerbated this situation [6, 7]. It is estimated that approximately 40 million children missed their measles routine vaccination in 2020–2022, posing significant challenges in maintaining and accelerating measles elimination and control worldwide.

Over the years, China has made considerable progress toward measles elimination through the combination of the National Expanded Program on Immunization (EPI) launched in 1978 and a series of nationwide and provincewide supplementary immunization activities (SIAs) implemented from 2003 to 2010 [8]. The success of these policies is evidenced by a continuous decrease in measles incidence from 1977 to 2020. The highest incidence rate (450.9/100 000) was reported before 1977, followed by a decrease in 1978–2009 (31.2/100 000) and a further decrease in 2009–2021 (1.6/100 000) [9]. In recent years, progress toward measles elimination has been impeded by population immunity gaps. The population immunity level required to eliminate measles remains uncertain due to a combination of factors, including undervaccination, suboptimal vaccine responses, and waning immunity after vaccination [10–12]. Additionally, the increased probability of measles importation from outside China, particularly after pandemic-related disruptions in routine measles-containing vaccine (MCV) uptake worldwide, presents a growing concern [13, 14]. Given the high transmissibility of the measles virus, even a single imported case can cause large outbreaks in underprotected communities [1]. Therefore, a timely quantification of measles-specific immunity levels by age in the Chinese population is urgently needed.

Modeling approaches are some of the main tools used to investigate measles immunity gaps in the Chinese population, allowing researchers to infer population susceptibility levels from historical case surveillance and child vaccination data [15–19]. While serological surveys constitute a more direct approach to estimating susceptibility levels, their use in the Chinese context has primarily been in response to measles outbreaks or SIA implementations [20–22]. Most of these existing serological surveys are outdated and are not representative of current measles immunity levels in Chinese settings with low endemicity of the measles virus or where SIAs have been absent for more than 10 years. Moreover, current estimates of population immunity levels rely on the simplistic assumption of homogeneous mixing in the population, disregarding the effect of the convolution of age-specific immunity levels and contact patterns in measles transmission [23, 24].

This study aims to quantify measles population immunity level by age and explore the factors contributing to heterogeneities in population immunity levels across different population groups and time periods. Specifically, we estimated both unadjusted immunity levels and contact-adjusted immunity levels, accounting for age-specific contact and immunity patterns. Given disparities in health resource distribution between rural and urban areas of China [25] and the ongoing effort toward measles elimination [26, 27], we decided to focus on a rural area. Specifically, a cross-sectional serological survey was conducted in 2021 among all age groups residing in Anhua County, Hunan Province, a typical rural area of China [28, 29]. Anhua County provides a relevant study setting due to a relatively high MCV coverage since 2000 and significant population immunity heterogeneity shaped by natural measles transmission, routine vaccination, and a series of provincewide SIAs targeting children aged between 8 months and 14 years in 2009–2010. Understanding these dynamics in a specific rural context is crucial for tailoring effective localized immunization strategies.

## METHOD

### Study Design and Participants

We conducted a cross-sectional, population-based serological survey from 24 June to 31 October 2021, in 3 townships (Qiangtang, Tianzhuang, and Jiangnan) of Anhua County, Hunan Province, China. Participants were eligible if they had resided in the study area for at least 3 months. Based on a predefined power of 95% and reported age-specific seroprevalence ranging from 58.5% to 97.3% in previous studies [20, 30], a sample size of 1015 participants aged 0–95 years was calculated using established methods for this population-based measles serology study. A 2-pronged approach was employed for participant recruitment. A random sample of households was selected from a list of all residents at the study site. Approximately 50 households were randomly selected from each village, and all eligible household members were included. Because the initial household-based enrollment did not meet the predefined sample size for child participants, the recruitment strategy was expanded to include school-age children. This supplemental school-based sample was not proportionally weighted to the household sample; rather, students were enrolled until the target sample size for children was reached. Within the 6 selected schools (2 primary schools, 3 middle schools, and 1 high school), classes and students were randomly selected and invited to participate. This approach ensured sufficient statistical power for analyzing child immunity. To verify that this recruitment strategy did not introduce biases excluding children not enrolled in formal education, we compared enrollment rates of the study participants and those reported in official records and found no substantial differences (Supplementary Table 1).

Trained nurses collected 2-mL venous blood samples from participants younger than age 6 years old and 4 mL from those aged 6 years and older. An anonymized questionnaire was conducted to collect participants’ demographic characteristics (birth date, sex, residence duration, education, income, occupation, and underlying conditions) and social contact behaviors (daily contact numbers, contact type, frequency, location, etc.). Details on the contact survey are reported in Liang et al [31]. Trained investigators obtained vaccination records for children younger than age 10 years. Two authors (Z.X. and Z.S.) manually extracted and double-entered the data in Excel 2021. To analyze the impact of vaccination programs, participants were categorized into 4 birth cohorts based on key vaccination milestones: (1) prevaccine period (born before 1965), (2) pre-EPI period (born 1965–1977, MCV introduction), (3) EPI period (born 1978–2009, national EPI implementation), and (4) post-SIAs period (born after nationwide SIAs in 2010).

### Laboratory Procedure

Anti-measles virus immunoglobulin G (IgG) antibodies were quantified using commercial enzyme-linked immunosorbent assay (ELISA) kits (SERION ELISA classic measles virus IgG, Institut Virion/Serion GmbH, Wurzburg, Germany). All samples were diluted 1:100 in assay diluent according to the manufacturer's instructions. The negative control and standard sera provided within the kit were used without further dilution. The resulting optical density values were converted into concentration units (mIU/mL) based on a calibration curve generated from standard serum using SERION software. As an established ELISA-based protective threshold for measles immunity is not yet available, we adopted a commonly accepted threshold of 200 mIU/mL in the primary analysis and a more conservative threshold of 300 mIU/mL in the sensitivity analysis. The ELISA used in this study was validated using the same laboratory protocol published elsewhere [32, 33], showing 98.9% sensitivity and 88.9% specificity, consistent with the “gold standard” plaque reduction neutralization test [32]. Participants with antibody concentrations exceeding the chosen protective threshold were considered seropositive/protective.

### Statistical Analysis

**Figure 1.**
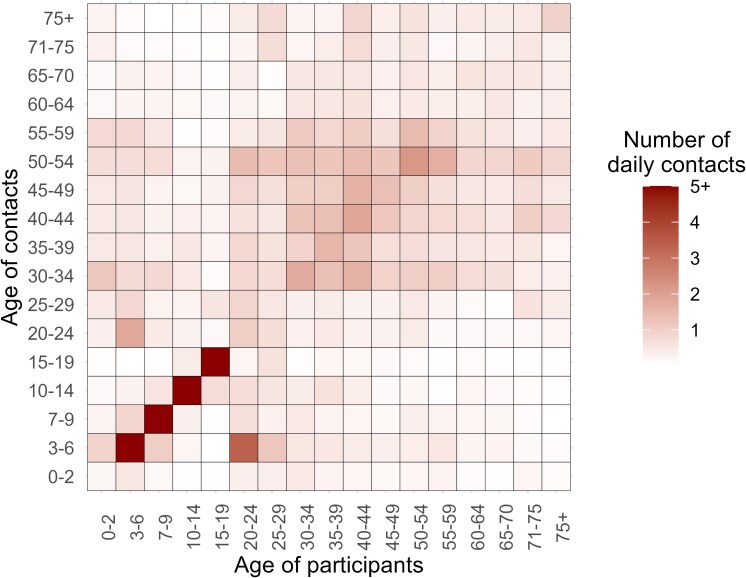
Contact matrix by age. Each cell represents the mean daily contact numbers that an individual in a specific age group has with others. The color intensity corresponds to the number of contacts. The contact matrix is based on postpandemic contact data for the for Hunan Province, China, reported in Liang et al [31].

We described the baseline characteristics of the participants and then estimated the age-specific geometric mean concentrations (GMCs) and seroprevalence, along with their 95% confidence intervals (95% CI). Generalized Additive Models, a statistical technique allowing for flexible modeling of nonlinear relationships, were used to fit measles-specific antibody concentration and seroprevalence by age. Given the heterogeneity in contacts relevant for measles virus transmission between different age groups, we additionally estimated contact-adjusted immunity using the methodology described in Funk et al [23]. Briefly, this approach adjusts the population's immunity level by incorporating age-specific mixing patterns. Namely, instead of assuming that all individuals contribute equally to transmission, the contact-adjusted immunity measure accounts for differences in contact frequency between age groups, expressed as $c_{ij}\frac{N_{i}}{N_{j}}$, where $c_{ij}$ represents the mean number of contacts that an individual of age j has with individuals of age i, weighted by the population sizes of these age groups ($N_{i}$ and $N_{j}$, respectively). Individuals with large number of contacts (eg, school-age individuals) and low immunity levels (eg, unvaccinated children) would have a larger contribution to the transmission, which is relevant to determine the herd immunity level of the population. The contact matrix by age was estimated using contact survey data collected from the study participants in the present study (Figure 1; more details are reported in Liang et al [31]). The corresponding mathematical details are provided in the Supplementary Text. It is important to stress that this procedure provides a populationwide estimate of effective immunity level, functioning similarly to a weighted average; as such, this approach does not inherently support age-specific estimations.

To investigate the associations of log-transformed antibody concentrations with birth cohorts, sex, underlying medical conditions, and socioeconomic status, we performed a multiple linear regression analysis. Additionally, to evaluate the impact of vaccination strategies on antibody concentrations, we employed interrupted time series regression, a statistical method suitable for analyzing the effect of interventions on time series data [34]. This approach allowed us to assess both the short-term (ie, immediate effect) and long-term (ie, average effect over time) changes in mean antibody concentration attributable to specific vaccination programs. We considered 3 vaccination strategies, including MCV vaccination, EPI, and SIA programs introduced in 1965 ($t_{MCV}$), 1978 ($t_{EPI}$), and 2010 ($t_{SIA}$), respectively. We additionally calculated the geometric mean ratio (GMR) by exponentiating the coefficient of each independent variable in the model. The GMR quantifies the fold-change in antibody concentration associated with a 1-unit increase in the corresponding independent variable. The corresponding method details are provided in the Supplementary Text. Furthermore, we compared long-term trends in log-transformed antibody concentrations across ages between individuals born in the prevaccine period and those born after the implementation of SIAs (>99% MCV coverage). This comparison was performed using both log-linear and exponential regression models. All statistical tests were 2-sided with a significance threshold of alpha = 0.05. All analyses were performed in R, version 4.1.0.

### Patient Consent Statement

This study was approved by the institutional review board of School of Public Health, Fudan University (IRB#2020-11-0857; #2020-11-0857-S; #2022-02-0950). We obtained written informed consent from all adults themselves or from caregivers of children aged younger than 18 years.

## RESULTS

**Figure 2.**
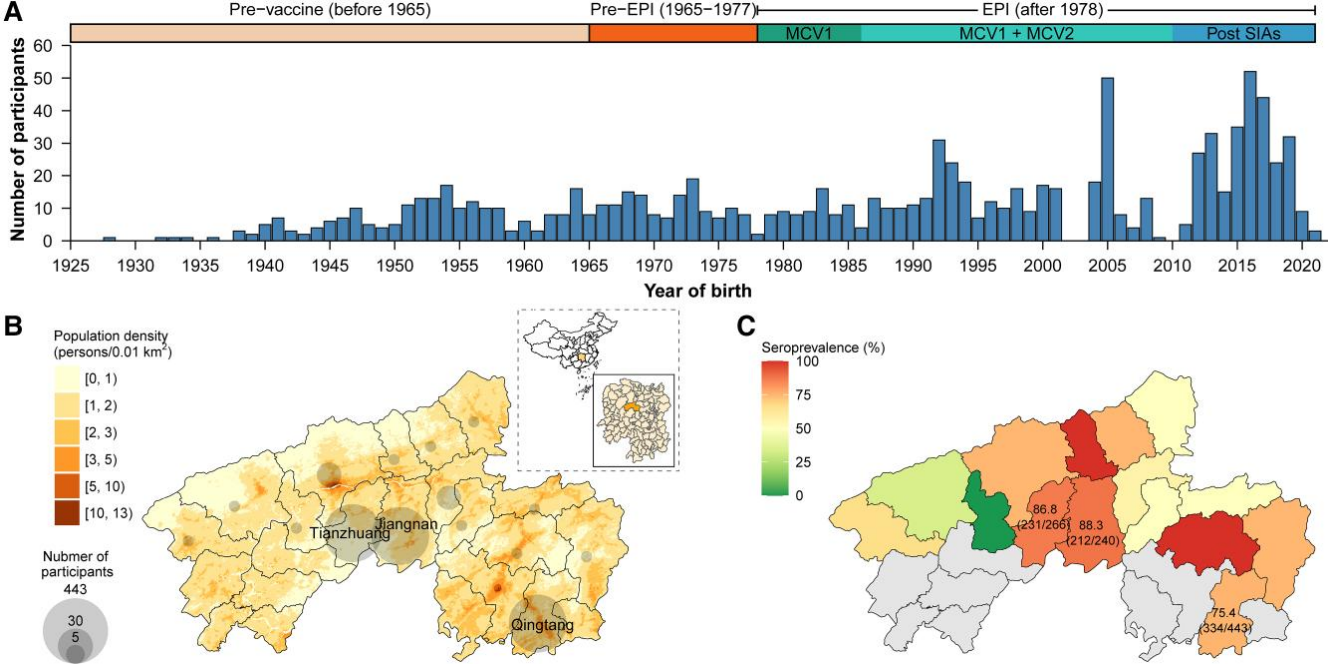
Temporal and geographical distribution of study participants and estimated seroprevalence. *A*, Number of study participants by year of birth. *B*, Geographical distribution of study participants. *C*, Estimated seroprevalence by township.

Our study enrolled a total of 1015 participants aged 0–95 years between 24 June and 31 October 2021 (as detailed in Figure 2*A* and Table 1). Local residents comprised the majority of participants (n = 951, 93.7%), with the remaining participants (n = 64, 6.3%) being children residing in other townships within Anhua County who were recruited through school-based enrollment at schools located within Anhua County (Figure 2*B*). The participant population exhibited a female predominance (60.5%, n = 614) of the total participants. The median age of participants was 29 years (interquartile range: 9–52 years). Notably, the age distribution of our study population deviates from that of the general population in the study area due to the intentional oversampling of children within the study design (29.5% vs 20.2% in the general population, *P* < .001). In terms of birth cohorts categorized by vaccination program implementation timelines, the study population included 209 individuals (20.6%) born in the prevaccine period, 141 individuals (13.9%) born in the pre-EPI period, 386 individuals (38.0%) born during the EPI period, and 279 individuals (27.5%) born after the implementation of SIAs. Among the 171 participants aged younger than 10 years with known vaccination records (171 of 252, representing 67.9%), the median age at the time of the first and second MCV doses was 8.5 months (interquartile range: 8.1–8.8 months) and 18.4 months (interquartile range: 18.1–18.9 months), respectively.

**Table 1.** Participants’ Characteristics

Characteristics	Overall (N = 1015)
**Sex**	…
Male	401 (39.5)
Female	614 (60.5)
**Age (y)**	…
Median (interquartile range, IQR)	29 (9–52)
0–9	252 (24.8)
10–19	121 (11.9)
20–29	134 (13.2)
30–39	126 (12.4)
40–49	94 (9.3)
50–59	108 (10.6)
60–69	91 (9.0)
≥70	89 (8.8)
**Birth period**	…
Prevaccine (1952–1964)	209 (20.6)
Pre-EPI (1965–1977)	141 (13.9)
EPI (1978–2009)	386 (38.0)
Post-SIAs (2010–2021)	279 (27.5)
**Underlying disease**	…
Yes^a^	233 (23.0)
Cardiocerebrovascular disease	93 (39.9)
Diabetes	23 (9.9)
Chronic respiratory disease	14 (6.0)
Others^b^	116 (49.8)
No	762 (75.1)
Missing	20 (2.0)
**Socioeconomic status^c^**	**…**
Low	329 (32.4)
Middle	406 (40.0)
High	250 (24.6)
Missing	30 (3.0)
**Local residency durations**	…
0–5 y	198 (19.5)
6–10 y	119 (11.7)
>10 y	693 (68.3)
Unknown	5 (0.5)
**Vaccination history**	…
Children with vaccination cards	171 (67.9)
Age at primary vaccination (median, IQR)	8.5 (8.1–8.8)
Age at booster vaccination (median, IQR)	18.4 (18.1–18.9)

Abbreviations: EPI, National Expanded Program on Immunization; SIA, supplementary immunization activity.

^a^This may not total 100% as 1 individual may have multiple concurrent diseases.

^b^This includes chronic liver disease, chronic kidney disease, tumor, and other diseases.

^c^The socioeconomic status index is calculated using education and occupation given data availability.

**Figure 3.**
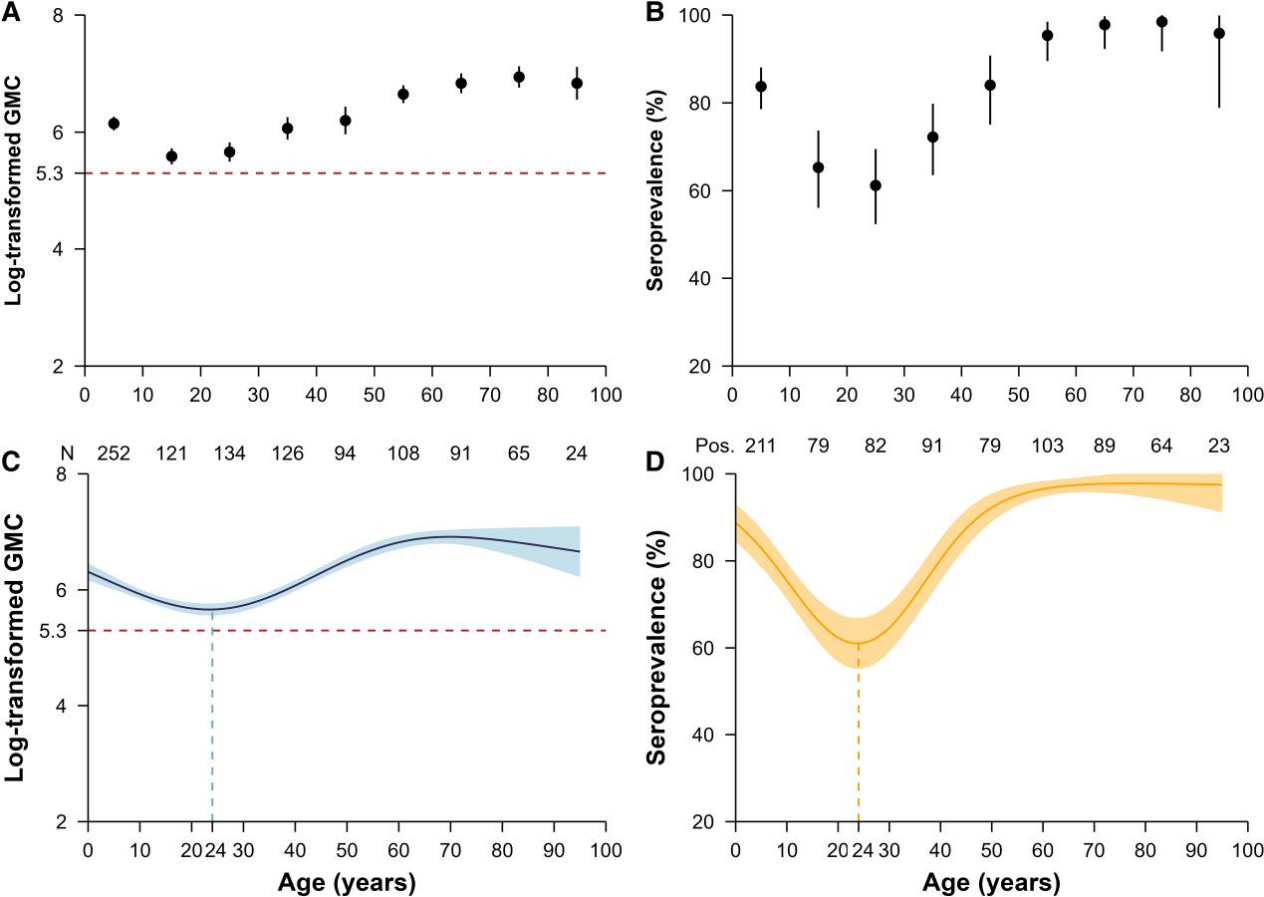
Measles-specific immunity levels by age. *A*, Observed and (*C*) predicted log-transformed geometric mean concentration (GMC); (*B*) observed and (*D*) predicted seroprevalence. A threshold of 200 mIU/mL was used to define seroprevalence. The points in (*A*) and (*B*) refer to the observed log-transformed GMC and seroprevalence, respectively. The thick curve in (*C*) and (*D*) is the predicted mean log-concentrations and seroprevalences from the generalized additive models. In (A-D), error bars and shaded areas show 95% confidence intervals. In the bottom of (A-B), the “N” and the numbers denote the sample size in each age group. The abbreviation “Pos.” refers to the number of seropositive participants.

We estimated an overall seroprevalence of 80.9% (821/1015; 95% CI, 78.3–83.3), with variations observed across geographic areas and age groups (Figure 2*C*). Comparable population immunity levels were observed in Jiangnan (seroprevalence 88.3% [212/240]; 95% CI, 83.6–92.1; mean log concentration 6.4) and Tianzhuang towns (86.8% [231/266]; 95% CI, 82.2–90.7; mean log concentration of 6.5) (*P* = .709). In contrast, Qingtang town exhibited a lower seroprevalence of 75.4% (334/443), 95% CI, 71.1–79.3, with a mean log concentration of 6.0 (*P* <.001) (Figure 2*C*). A significantly lower seroprevalence of 73.6% (95% CI, 69.9–77.3; mean log concentration 5.9) was estimated for individuals aged younger than 30 years compared with the 96.8% seroprevalence (95% CI, 94.7–98.9; mean log concentration of 6.8) observed among participants aged 50–69 years or older (Figure 3*A-B*). Notably, the age-specific seroprevalence in the 30–59 year group demonstrated high variability (range: 72.2–95.3%) compared with both younger and older age groups. Furthermore, we observed no significant difference in antibody concentration levels between male (seroprevalence 80.3%; 95% CI, 76.1–84.1; mean log concentration of 6.2) and female participants (seroprevalence 81.3%; 95% CI, 78.0–84.3; mean log concentration of 6.2) ($\chi^{2}$= 0.092, *P* = .762) (Supplementary Figure 1). Based on age-specific seroprevalence levels and contact patterns in Anhua, we further estimated the contact-adjusted immunity level to be 66.8% (95% CI, 56.6–75.1).

The generalized additive model revealed a continuous decline in log antibody concentrations with increasing age among individuals younger than age 24 years (range: 5.7–6.3) (Figure 3*C-D*). Conversely, measles antibody levels remained consistent in adults aged 60 years and older who were born in the prevaccine period (mean log concentration: 6.8, range: 6.7–6.9). Our seroprevalence estimates corroborated these trends, demonstrating a continuous decline in immunity among individuals aged 24 years and younger (range: 61.0–88.7%) and relatively stable immunity among individuals 60 years and above (range: 96.5–97.8%) (Figure 3*C-D*). Notably, the selection of the protective threshold did not alter these estimates (Supplementary Figure 2).

Our multivariable analysis revealed significant period-specific differences in measles antibody concentrations. Individuals born during the prevaccine period exhibited the highest concentrations (*P* < .001) (Supplementary Table 2). Notably, factors such as age, underlying medical conditions, and socioeconomic status did not exert a statistically significant influence on antibody levels. Employing interrupted time series regression, we observed a substantially faster rate of antibody decline among individuals born after the introduction of the measles vaccine (post-SIA period) compared to those born in preceding periods. Specifically, the post-SIA group experienced a 22.1% annual change in antibody levels (GMR = 1.221; 95% CI, 1.162–1.284; *P* < .001). In contrast, individuals from the prevaccine period displayed a negligible change (0.2%, GMR = 0.998; 95% CI, 0.982–1.014; *P* = .783). The pre-EPI and EPI and SIA periods also demonstrated minimal decline rates (3.9%, GMR = 0.961; 95% CI, 0.917–1.006; *P* = .089) and (1.8%, GMR = 1.018; 95% CI, 0.970–1.068; *P* = .462), respectively (Table 2). This finding is further supported by the antibody trend test conducted over different age groups using either a log-linear model (coefficient for prevaccine versus post-EPI period: 0.001 [95% CI, −0.012 to 0.015; *P* = .885] versus −0.19 [95% CI, −0.23 to −0.14; *P* < .001]) or an exponential model (−0.003 [95% CI, −0.018 to 0.015; *P* = .669] versus −0.25 [95% CI, −0.34 to −0.18; *P* < .001)] (Supplementary Table 3).

**Table 2.** Interrupted Time Series Analysis of Measles Antibody Concentrations

Characteristics	Sample Size	Median Concentration (Q1, Q3)	Adjustedβ (95% CI)	Adjusted Geometric Mean Ratio (95% CI)	*P* Value
**Intercept**	**…**	-	6.853 (6.312, 7.393)	946.282 (551.121, 1624.781)	<.001
**Birth period**	**…**	…	…	…	
Prevaccine (1925–1964)	209	942.2 (560.2, 1678.8)	…	…	
Birth cohort-specific Ab trend	…	-	−0.002 (−0.018, 0.014)	0.998 (0.982, 1.014)	.783
Pre-EPI (1965–1977)	141	637.6 (370.1, 1341.0)	…	…	
Ab-level change postvaccine introduction (1965)	…	-	−0.179 (−0.557, 0.200)	0.836 (0.573, 1.221)	.354
Birth cohort-specific Ab trend change (1966–1977)	…	-	−0.040 (−0.086, 0.006)	0.961 (0.917, 1.006)	.089
EPI and SIAs (1978–2009)	386	334.8 (160.9, 633.8)	…	…	
Ab-level change post EPI introduction (1978)	…	-	0.015 (−0.415, 0.444)	1.015 (0.660, 1.559)	.947
Birth cohort–specific Ab trend change (1979–2009)	…	-	0.018 (−0.030, 0.066)	1.018 (0.970, 1.068)	.462
Ab-level change post-SIAs introduction (1995–2009)	…	-	0.147 (−0.201, 0.495)	1.158 (0.818, 1.640)	.408
Post-SIAs (2010–2021)	279	415.5 (237.6, 812.9)	…	…	
Ab-level change without boosting from SIAs (2010)	…	-	−0.236 (−0.821, 0.349)	0.790 (0.440, 1.417)	.428
Birth cohort-specific Ab trend change (2011–2021)	…	-	0.200 (0.150, 0.250)	1.221 (1.162, 1.284)	**<0**.**001**
**Sex**	**…**	…	…	…	
Male	401	514.1 (247.5, 974.8)	Reference	Reference	-
Female	614	465.3 (254.1, 968.0)	0.048 (−0.071, 0.167)	1.049 (0.931, 1.181)	.432
**Any underlying disease**	**…**	…	…	…	
Yes	233	748.0 (369.4, 1463.1)	−0.054 (−0.221, 0.113)	0.947 (0.802, 1.119)	.525
No	762	440.7 (222.2, 869.6)	Reference	Reference	-
Unknown	20	232.2 (132.1, 328.6)	−0.427 (−0.840, −0.013)	0.653 (0.432, 0.987)	.**043**
**Socioeconomic status**	**…**	…	…	…	
Low	329	414.3 (240.2, 757.2)	Reference	Reference	-
Middle	406	581.7 (311.1, 1262.1)	0.090 (−0.188, 0.368)	1.094 (0.828, 1.445)	.527
High	250	422.0 (183.4, 896.1)	0.112 (−0.180, 0.403)	1.118 (0.835, 1.497)	.452
Missing	30	508.5 (319.5, 1477.1)	0.249 (−0.176, 0.675)	1.283 (0.838, 1.964)	.250

Abbreviations: Ab, antibody; CI, confidence interval; EPI, National Expanded Program on Immunization; Q, quartile; SIA, supplementary immunization activity.

## DISCUSSION

Leveraging measles IgG antibody measurements collected in southern China during 2021, this study assessed the current level of population immunity against measles within a general Chinese population, along with its variation across different age groups. We observed that despite achieving a commendable 99% coverage of the measles-containing vaccine since 2010, the study population's immunity level has not yet reached the critical threshold of 95%. Furthermore, our findings reveal heterogeneous patterns in both the initial level and subsequent decay of immunity among individuals born in distinct time periods. Notably, a significant immunity gap was identified specifically within the post-SIA birth cohort, attributable to their demonstrably faster rate of immunity decline compared to other groups.

Our 2021 estimates for both overall (80.9%; 95% CI, 78.3–83.3) and contact-adjusted (66.8%; 95% CI, 56.6–75.1) measles immunity levels indicate that the current level of population immunity is inadequate to prevent measles virus transmission [35]. These findings aligned with reports from other Chinese provinces during 2020–2023, which reported immunity levels ranging from 71% to 94% [20, 36–39]. Despite regional heterogeneity in China, almost all studies have observed a similar U-shaped distribution of age-specific immunity, with relatively higher seroprevalence and GMCs in young children and older adults. Additionally, we observed a significant geographic variation in seroprevalence, with Qingtang showing a notably lower seroprevalence (75.4%) compared to Jiangnan (88.3%) and Tianzhuang (86.8%). One possible explanation is the difference in healthcare accessibility due to the geographical distance between local residences and hospitals. Qingtang, characterized by more difficult terrain and mountainous landscapes, may face greater challenges in healthcare delivery compared to the other 2 towns. This study demonstrates heterogeneous patterns of antibody waning among individuals born at different points relative to the implementation of various vaccination programs. Notably, individuals born in the prevaccine period exhibited minimal changes in antibody levels, likely reflecting the persistent nature of measles-specific antibodies acquired through natural infection, as documented in prior research [40, 41]. Conversely, individuals born after the introduction of SIAs displayed substantial antibody waning. This phenomenon can be primarily attributed to the natural decline of vaccine-induced antibodies without subsequent boosting from measles infections [32, 42]. Long-term antibody waning following routine vaccinations has been established as a significant factor contributing to measles outbreaks in China and elsewhere [42, 43].

We estimated a 15.8% increase in antibody levels among the birth cohorts targeted by the 1995–2009 SIA (GMR = 1.158; 95% CI, 0.818–1.640; *P* = .408). It is important to stress that this result was not statistically significant, possibly due to the small sample size and individual heterogeneity. However, this estimate is consistent with a house-to-house survey conducted in 2013, which reported a 10.0% to 17.6% increase in measles seroprevalence among already vaccinated children [44]. This finding, combined with a previously reported 8.8% to 16.8% increase in MCV1 coverage among unvaccinated children due to SIAs [44], underscores the importance of SIAs in fostering population immunity, particularly in unvaccinated children and vaccinated individuals with waning immunity. Our findings also indicate that seroprevalence among individuals aged 10–19 years (65.3% [95% CI, 56.1–73.7]) and 20–29 years (61.2% [95% CI, 52.4–69.4]) is markedly lower than the herd immunity threshold for measles (typically around 95%). Given that these age groups also exhibit higher contact rates (Figure 1), their lower immunity levels may pose a substantial challenge to measles elimination efforts, increasing the risk of local outbreaks especially within these population groups. These findings emphasize the need for the design of future regionwide or nationwide measles elimination programs. Achieving and sustaining a 95% population immunity threshold solely through increasing routine vaccination coverage to 99% does not appear to be feasible due to the waning immunity observed in postvaccine era individuals. This critical factor, together with the estimate immunity gaps by age, call for the careful design and implementation of future SIA strategies. Further research is warranted to design effective immunization plans while accounting for population immunity gaps, demographic shifts, and natural infection dynamics [45].

This study has a set of limitations. First, given the intrinsic characteristics of our cross-sectional study design, we are unable to longitudinally explore individual antibody changes with age. However, our conclusions regarding differences in population immunity over time and subsequent waning rates, driven by time-specific measles levels in the population and vaccination policies, align with recently reported data [43, 46]. Additionally, while it is plausible that individuals born earlier may have been reexposed more recently, we could not quantify the contribution of each infection or vaccination event to changes in individual/population antibody levels. Natural reexposure likely plays a role in sustaining immunity among older cohorts, contrasting with younger cohorts, whose immunity primarily depends on vaccination and may wane over time in the absence of natural boosting. This limitation arises from having antibody measurements from a single time point, highlighting the need for further research using longitudinal study designs and/or mechanistic models to infer lifelong immunity trajectories shaped by vaccination and infection events. Second, the limited availability of individual vaccination records, along with potential recall bias, hamper our ability to perform further analyses on the association between individual vaccine uptake and the observed immunity waning pattern. However, the former would not affect our major conclusion, as previous study has already shown consistent antibody waning rate across MCV doses [33]. Third, seasonal or regional variations in measles exposure, not captured during the study period, may have influenced naturally acquired immunity level. Fourth, as this study was conducted in a rural population, caution should be taken when extrapolating the findings to urban settings, where improved healthcare infrastructure and higher vaccination rates may result in differing immunity dynamics. Last, ELISA cannot distinguish between functional and nonfunctional antibodies, which may lead to an overestimation of the proportion of individuals with low or absent protective immunity. Although there is some debate regarding the level of the protective threshold ELISA value for measles (set here at 200 mIU/mL), this uncertainty does not affect our finding regarding the age-specific immunity profile (Supplementary Figure 2).

In conclusion, this study underscores the discrepancy between high local MCV uptake rates and the overall population immunity level. While routine immunization programs effectively protect young infants and children during their formative years, they may not be sufficient to maintain a sustained 95% population immunity. Additionally, the ongoing threat posed by postvaccination immunity waning necessitates the implementation of dynamic surveillance systems for measles population immunity, particularly in settings where the virus is not actively circulating.

## Supplementary Material

ofaf216_Supplementary_Data

## Supplementary Data

Supplementary materials are available at *Open Forum Infectious Diseases* online. Consisting of data provided by the authors to benefit the reader, the posted materials are not copyedited and are the sole responsibility of the authors, so questions or comments should be addressed to the corresponding author.

## Notes

***Acknowledgments.*** The authors thank all contributors and study participants for their participation The computations in this research were performed using the CFFF platform of Fudan University.

***Author Contributions.*** H.Y. conceived the study. Q.W., X.Z., J.Z., S.Z., and Y.L. participated in data collection. Q.W., S.Z., and X.Z. did the laboratory tests. Y.W., Q.W., and W.W. analyzed the data, prepared figures and the first draft of the manuscript. P.W., F.T., M.A., and H.Y. commented on the data and their interpretation and critically revised the content.

***Financial support.*** This project was supported by the Shanghai Municipal Science and Technology Major Project (grant number ZD2021CY001 to H.Y.), the Key Program of the National Natural Science Foundation of China (grant number 82130093 to H.Y.), the Young Scientists Fund of the National Natural Science Foundation of China (grant number 82304205 to W.W.), and the National Natural Science Foundation of China (grant numbers 92369118 and 82304202 to J.Z.). The funders had no role in study design, data collection and analysis, decision to publish, or preparation of the manuscript.
