# Supplementary material for: Measles Population Immunity in Hunan, China: A Serological Assessment
Source: Open Forum Infect Dis. 2025 Apr 8;12(5):ofaf216. doi: 10.1093/ofid/ofaf216 (PMC12039485; doi:10.1093/ofid/ofaf216)
Supplement: ofaf216_Supplementary_Data [file ofaf216_supplementary_data.docx]

# Supplementary Note 1. Estimation of contact-adjusted immunity level

From the mathematical standpoint, the contact-adjusted immunity, $r'$, can be estimated as follows:

$$r'=1-\frac{\rho\left( c_{ij}\frac{N_{i}}{N_{j}}(1-r_{i}) \right)}{\rho\left( c_{ij}\frac{N_{i}}{N_{j}} \right)}$$

where $\rho\left( X \right)$ denotes the largest eigenvalue of matrix $X$, $c_{ij}$ is the mean contact numbers that an individual of age $j$ has with individuals of age$i$, $N_{i}$ is the number of individuals in age group $i$, $r_{i}$ is the seroprevalence in age group $i$. The contact matrix by age was estimated using contact survey data collected from the study participants in the present study (Figure1; more details are reported in Liang et al. [1]). The formula is inspired by the relationship between the effective reproductive number $R_{e}$ and the basic reproductive number $R_{0}$, where $R_{e}=\left( 1-r^{'} \right)R_{0}$, with both $R_{e}$ and $R_{0}$ being calculated as the largest eigenvalue of the next-generation matrix, which was constructed based the contact matrix. A detailed derivation can be found in Funk et al.’s work [2]. The 95th percentile bootstrap confidence intervals were determined to quantify the uncertainty for the age-specific seroprevalence and contact matrix.

# Supplementary Note 2. Using interrupted time series regression to evaluate the impact of vaccination strategy

The regression model was defined as follows:

$Y=\beta_{0}+\beta_{1}*t+\beta_{2}*MCV\_status+\beta_{3}*{(t-t_{MCV})}_{+}+\beta_{4}*EPI\_status+\beta_{5}*{(t-t_{EPI})}_{+}$+

$\beta_{6}*SIA\_status+\beta_{7}*\left( t-t_{SIA} \right)_{+}+ \beta_{8}*Sex+ \beta_{9}*Underlying\_conditions+\beta_{10}*SES+\varepsilon$

where *Y* represents the individual’s log-transformed antibody concentration, and *t* denotes the time difference between the individual’s birth time and the earliest birth year in the study (1926). For the three vaccination strategies, $\beta_{2}$, $\beta_{4}$, and $\beta_{6}$ denote their impact on instant level change in antibody concentration, while $\beta_{3}$, $\beta_{5}$, and $\beta_{7}$ measure their impact on mean antibody concentration as time increases. $MCV\_status$ and $EPI\_status$ indicate whether MCV or EPI introduction occurred at the time *t*; $SIA\_status$ indicates whether individual immune boosting occurs through SIAs implemented in 2009-2010, targeting birth cohorts from 1995 to 2009. The functions ${(t-t_{MCV})}_{+}$, ${(t-t_{EPI})}_{+}$, and ${(t-t_{SIA})}_{+}$ are used to generate time series data starting from the corresponding intervention launched, which are defined as:

${(t-t_{MCV})}_{+}$= $t-t_{MCV}$ if $t$>$t_{MCV}$

${(t-t_{EPI})}_{+}=$ $t-t_{EPI}$if $t$>$t_{EPI}$

${(t-t_{SIA})}_{+}$=$t-t_{SIA}$ if $t$>$t_{SIA}$

Otherwise, they equal 0. *Sex,* $Underlying\_conditions$, and $SES$ indicate individual sex, history of underlying medical conditions, and socioeconomic status, respectively.

# Supplementary Tables

**Table S1. The school enrollment rates for School-age children**

| **Age group (years)** | **School enrollment rates (%, students / total) ^a^** | |
| --- | --- | --- |
|  | Study participants | Population in Yiyang city ^b^ |
| 3-9 | 90.4% (206/228) | 93.2% (322,751/346,256) |
| 10-14 | 100% (21/21) | 99.6% (227,594/228,479) |
| 15-19 | 98.7% (74/75) | 99.8% (164,685/165,052) |

^a^ Including kindergarten, primary school, junior high school, senior high school, and higher education.

^b^ As statistical data for Anhua County is unavailable, we reported data from Yiyang City [3], which Anhua County is a part of.

**Table S2.** **Multivariable analysis of measles antibody concentrations.**

| **Characteristics** | **N** | **Median concentration**  **(Q1, Q3)** | **Adjusted**$\beta$  **(95% CI)** | **Adjusted geometric mean ratio (95% CI)** | **P-value** |
| --- | --- | --- | --- | --- | --- |
| **Intercept** |  | - | 6.674 (6.360, 6.988) | 791.633 (578.157, 1083.932) | **<0.001** |
| **Birth period** |  |  |  |  |  |
| Pre-vaccine (1925-1964) | 209 | 942.2 (560.2, 1678.8) | Reference | Reference | **-** |
| Pre-EPI (1965-1977) | 141 | 637.6 (370.1, 1341.0) | -0.444 (-0.649, -0.239) | 0.641 (0.523, 0.788) | **<0.001** |
| EPI & SIAs (1978-2009) | 386 | 334.8 (160.9 ,633.8) | -1.05 (-1.242, -0.858) | 0.350 (0.289, 0.424) | **<0.001** |
| Post-SIAs (2010-2021) | 279 | 415.5 (237.6 ,812.9) | -0.615 (-0.945, -0.286) | 0.540 (0.389, 0.751) | **<0.001** |
| **Sex** |  |  |  |  |  |
| Male | 401 | 514.1 (247.5, 974.8) | Reference | Reference | - |
| Female | 614 | 465.3 (254.1, 968.0) | 0.037 (-0.085, 0.16) | 1.038 (0.918, 1.173) | 0.551 |
| **Any underlying disease** |  |  |  |  |  |
| Yes | 233 | 748.0 (369.4, 1463.1) | -0.005 (-0.172, 0.163) | 0.995 (0.842, 1.177) | 0.957 |
| No | 762 | 440.7 (222.2, 869.6) | Reference | Reference | - |
| Unknown | 20 | 232.2 (132.1, 328.6) | -0.649 (-1.071, -0.227) | 0.523 (0.343, 0.797) | **0.003** |
| **Socioeconomic status** |  |  |  |  |  |
| Low | 329 | 414.3 (240.2, 757.2) | Reference | Reference | - |
| Middle | 406 | 581.7 (311.1, 1262.1) | 0.183 (-0.098, 0.464) | 1.201 (0.907, 1.591) | 0.202 |
| High | 250 | 422.0 (183.4, 896.1) | 0.224 (-0.066, 0.513) | 1.251 (0.936, 1.671) | 0.130 |
| Missing | 30 | 508.5 (319.5, 1477.1) | 0.406 (-0.021, 0.833) | 1.501 (0.979, 2.301) | 0.063 |

**Table S3. Trend tests for measles antibody concentration changes over ages.**

| Parameter | **Pre-vaccine stage**  (N = 209) | | | | **Post-SIAs stage**  (N = 279) | | | |
| --- | --- | --- | --- | --- | --- | --- | --- | --- |
|  | Log-linear model | | Exponential model | | Log-linear model | | Exponential model | |
|  | $\beta$(95% CI) | P-value | $\beta$ (95% CI) | P-value | $\beta$(95%CI) | P-value | $\beta$ (95%CI) | P-value |
| Intercept | 6.79  (5.85, 7.72) | **<0.001** | 1579.55  (611.33, 4201.54) | **0.040** | 7.11  (6.84-7.38) | **<0.001** | 2276.68  (1592.97, 3230.97) | **<0.001** |
| Age | 0.001  (-0.012, 0.015) | 0.855 | -0.003  (-0.018, 0.015) | 0.669 | -0.19  (-0.23, -0.14) | **<0.001** | -0.25  (-0.34, -0.18) | **<0.001** |
| AIC | 488.98 | | 4111.09 | | 616.05 | | 3492.06 | |
| *R^2^* | 0.02% | | 0.09% | | 22.4% | | 15.8% | |

# Supplementary Figures


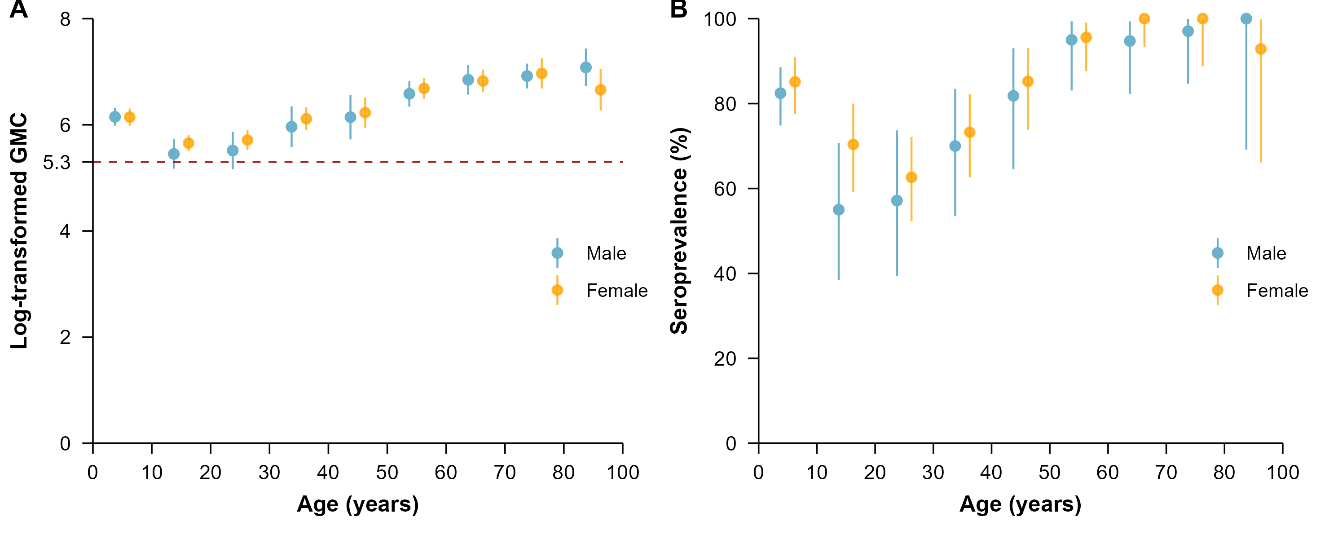


**Figure S1. Measles-specific immunity levels by age and sex.**

(**A**) observed age-specific log-transformed geometric mean concentration (GMC) among males and females; (**B**) observed age-specific seroprevalences among male and females; The points in the panel (A) and (B) refer to the observed log-transformed GMC and seroprevalence respectively. Error bars show 95% confidence intervals.


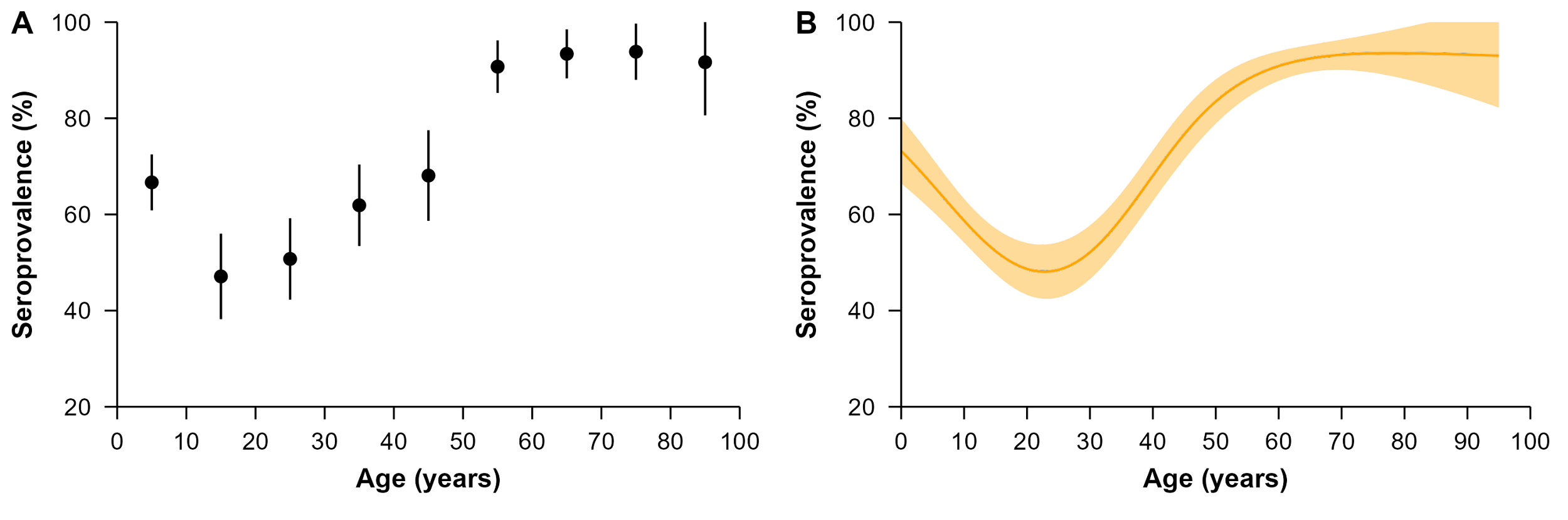


**Figure S2**. **Measles-specific immunity levels by age using a protective threshold of 300 mIU/ml**.

(**A**) observed and (**B**) predicted seroprevalence. The points and thick line in (A) and (B) refer to the observed and predicted mean seroprevalence. Error bars and shaded areas show 95% confidence interval.

# Reference

1. Liang Y, You Q, Wang Q, et al. Social contact patterns and their impact on the transmission of respiratory pathogens in rural China. Infectious Disease Modelling.2025; 10(2):439-452.

2. Funk S, Knapp JK, Lebo E, et al. Combining serological and contact data to derive target immunity levels for achieving and maintaining measles elimination. BMC Med.2019; 17(1):180.

3. Yiyang Municipal Bureau of Statistics.Yiyang Statistic Yearbook. [2025-02-20]. <http://tjj.yiyang.gov.cn/4948/38748/>.
